# SmedOB1 is Required for Planarian Homeostasis and Regeneration

**DOI:** 10.1038/srep34013

**Published:** 2016-09-22

**Authors:** Shanshan Yin, Yan Huang, Yingnan Zhangfang, Xiaoqin Zhong, Pengqing Li, Junjiu Huang, Dan Liu, Zhou Songyang

**Affiliations:** 1 Key Laboratory of Gene Engineering of the Ministry of Education, State Key Laboratory of Biocontrol and Institute of Healthy Aging Research, School of Life Sciences, Sun Yat-sen University, Guangzhou, China; 2 Verna and Marrs McLean Department of Biochemistry and Molecular Biology, Baylor College of Medicine, Houston, USA

## Abstract

The planarian flatworm is an emerging model that is useful for studying homeostasis and regeneration due to its unique adult stem cells (ASCs). Previously, planaria were found to share mammalian TTAGGG chromosome ends and telomerases; however, their telomere protection proteins have not yet been identified. In *Schmidtea mediterranea*, we identified a homologue of the human protection of telomeres 1 (POT1) with an OB-fold (SmedOB1). SmedOB1 is evolutionarily conserved among species and is ubiquitously expressed throughout the whole body. Feeding with SmedOB1 double-stranded RNAs (dsRNAs) led to homeostasis abnormalities in the head and pharynx. Furthermore, several ASC progeny markers were downregulated, and regeneration was impaired. Here we found that SmedOB1 is required for telomeric DNA-protein complex formation and it associates with the telomere TTAGGG sequence *in vitro*. Moreover, DNA damage and apoptosis signals in planarian were significantly affected by SmedOB1 RNAi. We also confirmed these phenotypes in *Dugesia japonica,* another flatworm species. Our work identified a novel telomere-associated protein SmedOB1 in planarian, which is required for planarian homeostasis and regeneration. The phylogenetic and functional conservations of SmedOB1 provide one mechanism by which planarians maintain telomere and genome stability to ensure their immortality and shed light on the regeneration medicine of humans.

Adult stem cells (ASCs) self-renew and give rise to multiple cell types during tissue homeostasis and regeneration^1^,^2^. Telomere maintenance is critical for ASC self-renewal and pluripotency^3^,^4^. Telomeres are G-rich tandem repeat sequences at the end of linear chromosomes. Maintenance of telomere length and integrity depends on the telomerase holoenzyme as well as a multitude of telomere-binding proteins^5^,^6^. Although the exact telomere DNA sequences and the specific telomeric proteins involved vary between organisms, many of the key telomere regulatory proteins are evolutionarily conserved. For example, the single-stranded telomere DNA (G-strand) binding protein POT1 is conserved from *Oxytricha nova* (TEBPα) to humans (POT1)^7^,^8^. POT1 contains oligonucleotide/oligosaccharide-binding folds (OB-folds) and is ubiquitously expressed. Deletion of the Pot1 gene in fission yeast causes the rapid loss of telomeric DNA, followed by chromosome instability and cell death^8^. POT1 homologs in C. elegans, CeOB1 and CeOB2, were shown to bind to the G-strand and C-strand overhang, respectively^9^. Mice have two Pot1 orthologues (Pot1a and Pot1b) with distinct functions in telomere maintenance and protection^10^. Human POT1 was originally cloned based on its sequence homology to TEBPα and is critical for ensuring proper telomere length control and stability^7^,^8^. Interestingly, a series of mutations in POT1 abolished its DNA binding ability, which is related to cutaneous malignant melanoma, and POT1 loss-of-function mutations cause predisposition to familial melanoma, suggesting that the telomere protection function of POT1 may ensure genome stability and prevent tumourigenesis^11^,^12^.

Freshwater planarians are well known for their remarkable regenerative abilities, in which lost body parts are regenerated from ASCs called neoblasts. Exploring how telomere regulation contributes to ASC maintenance in planarians should greatly advance our understanding of tissue regeneration in humans. The telomere sequences in planarians are similar to those in mammals (TTAGGG)^13^,^14^. Additionally, telomerase has been identified in *Schmidtea mediterranea* and is activated when asexual planarians split and regenerate^3^. However, the mechanisms by which planarians maintain telomeres and genome stability remain largely unknown. The regulatory proteins that participate in telomere protection and length control in planarians have yet to be identified.

Here, we present the identification and investigation of a mammalian POT1 homologue with a conserved OB fold (SmedOB1) in the planarian *Schmidtea mediterranea*. SmedOB1 is required for tissue homeostasis and regeneration. Inhibiting SmedOB1 expression may disrupt stem cell self-renewal causing DNA damage and apoptosis. By EMSA assays using purified proteins or endogenous nuclear extracts from planarians, we confirmed that SmedOB1 is required for telomeric DNA-protein complex association. Consistently, loss of SmedOB1 significantly increased the DNA damage as well as apoptosis signals in planarian. We also confirmed these phenotypes in another flatworm species, *Dugesia japonica*. In this study, we identified a novel telomere associated SmedOB1 protein that is required for freshwater planarian homeostasis and regeneration, indicating that this telomere-associated protein is crucial for planarians to maintain telomeres and genome stability to ensure their immortality.

## Results

### Identification of a *Schmidtea mediterranea* homologue of human POT1

**Figure 1:**
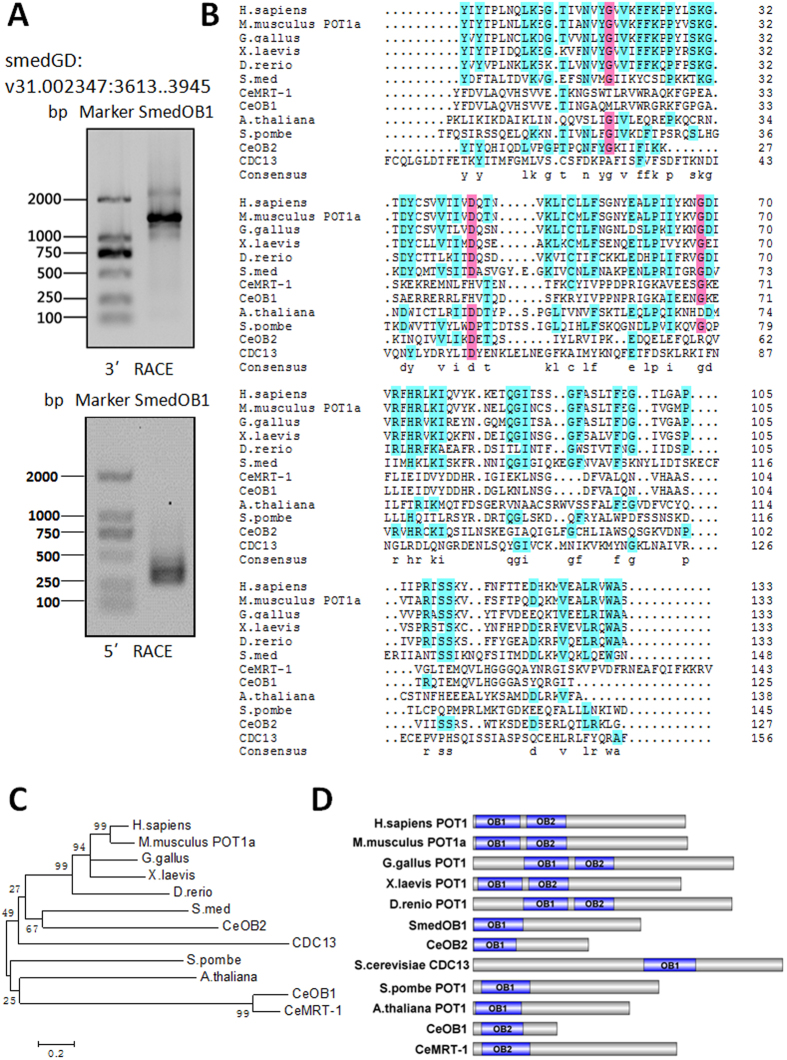
Identification of the human POT1 homologue SmedOB1 in *Schmidtea mediterranea.* (**A**) Primers based on the BLAST result (V31.002347: 3945..3613) were used for the 5′ RACE and 3′ RACE PCR of SmedOB1. (**B**) Sequence homology of the OB1 fold domain in POT1 or proteins similar to POT1 in human (H. sapiens POT1), mouse (M. musculus POT1a), chicken (G. gallus POT1), frog (X. laevis POT1), zebrafish (D. rerio POT1), *Schmidtea mediterranea* (S. med POT1, i.e. SmedOB1), Arabidopsis thaliana (A. thaliana POT1), yeast (S. pombe POT1, S. cerevisiae CDC13) and C. elegans (CeOB1, CeOB2 and CeMRT-1, the CeOB1 and CeMRT-1 have only OB2 domain and were analysed with OB1 of the others) as analysed using Cluster X. (**C**) A phylogenetic tree of the full-length POT1 proteins and relate proteins among species were constructed using Cluster X and MEGA4. (**D**) A schematic diagram of the OB fold domains (OB1 and OB2) in POT1 full-length proteins from various species was showed.

Planarians have telomere repeat sequences similar to those of humans (TTAGGG)^13^,^14^. Indeed, using probes containing TTAGGG sequences, we detected telomeres in metaphase spreads of two species of planarians, *Schmidtea mediterranea and Dugesia japonica* (Supplementary Fig. S1A,B). We then used the sequence from the longest isoform of human POT1 (isoform 1) to search the *Schmidtea mediterranea* genome database (http://smedgd.stowers.org) and found that the 333-bp genome sequence V31.002347: 3945..3613 was a significant match. Then, using 5′ and 3′RACE PCR, a 1506-bp full-length cDNA was obtained. We named this gene SmedOB1 (Fig. 1A) (Supplementary Fig. S2A,B).

The predicted protein has 501 amino acids with an N-terminal region (amino acids 1–147) that is highly conserved among other species (Fig. 1B,C). A domain analysis revealed the presence of one OB fold that corresponds to OB1 (Fig. 1D). Although both OB folds of the human POT1 possess ssDNA binding capacity, OB1 is indispensible for human POT1 binding to ssDNA. The C-terminus of human POT1 is responsible for associating with other shelterin complex protein such as TPP1^15^. Accordingly, we hypothesized that SmedOB1 may also be involved in ssDNA binding.

### SmedOB1 is required for tissue homeostasis in *Schmidtea mediterranea*

**Figure 2:**
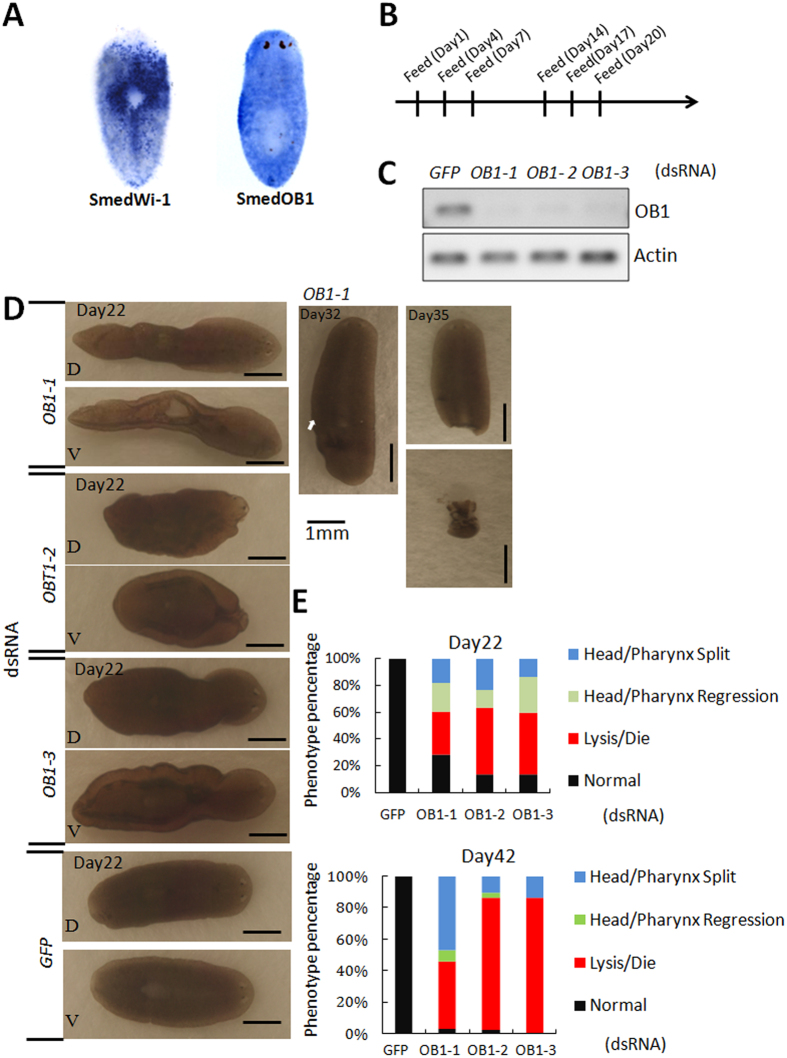
SmedOB1 is required for homeostasis in *Schmidtea mediterranea.* (**A**) WISH of SmedOB1 in planaria. SmedWi-1 was used as a positive control. (**B**) A flow diagram of the dsRNA-feeding assay. (**C**) RNAi efficiency after two rounds of dsRNA feeding. OB1-1, OB1-2 and OB1-3 are three different dsRNAs targeting SmedOB1. GFP RNAi is a negative control. (**D**) Phenotypes of planarians after feeding with SmedOB1 dsRNAs. Typical phenotypes are shown. (**E**) Results of statistical analysis of the phenotype ratios of planarians after feeding with SmedOB1 dsRNAs. Each group includes 30 worms.

To decipher the role of SmedOB1 in planaria, we first performed whole-mount *in situ* hybridization (WISH) analysis using SmedWi-1, an adult stem cell marker, as a positive control^16^. SmedOB1 was expressed ubiquitously in the whole body (Fig. 2A). To better understand the function of SmedOB1, we conducted RNAi experiments in intact *Schmidtea mediterranea* (Fig. 2B). The worms were fed double-stranded RNAs (dsRNAs) targeted against SmedOB1. The dsRNA targeting GFP served as a negative control. According to RT-PCR analysis, SmedOB1 was efficiently silenced after two rounds of dsRNA feeding (Fig. 2C). When we examined the RNAi-treated worms, we observed that the SmedOB1-RNAi planarians were morphologically abnormal compared with the control worms. The SmedOB1-RNAi planarians showed tissue injuries near the pharynx and behind the eyespots (darker regions in the body) (Fig. 2D and Supplementary Fig. S3A). Over time, these worms either split at the sites of these apparent tissue injuries or died, suggesting a critical role for SmedOB1 in planarian homeostasis (Fig. 2D, right, and Fig. 2E).

### SmedOB1 is required for regeneration in *Schmidtea mediterranea*

When planarians are irradiated with X-rays, the ASCs lose their ability to proliferate, and the worms die. Also the stem cell markers like SmedWi-1 will be decreased^17^. We therefore examined the expression level of SmedOB1 following X-ray irradiation. As shown in Supplementary Fig. S4, the expression level of SmedOB1 decreased to approximately 30% after irradiation (Supplementary Fig. S4), further linking SmedOB1 to the planarian regeneration process.

**Figure 3:**
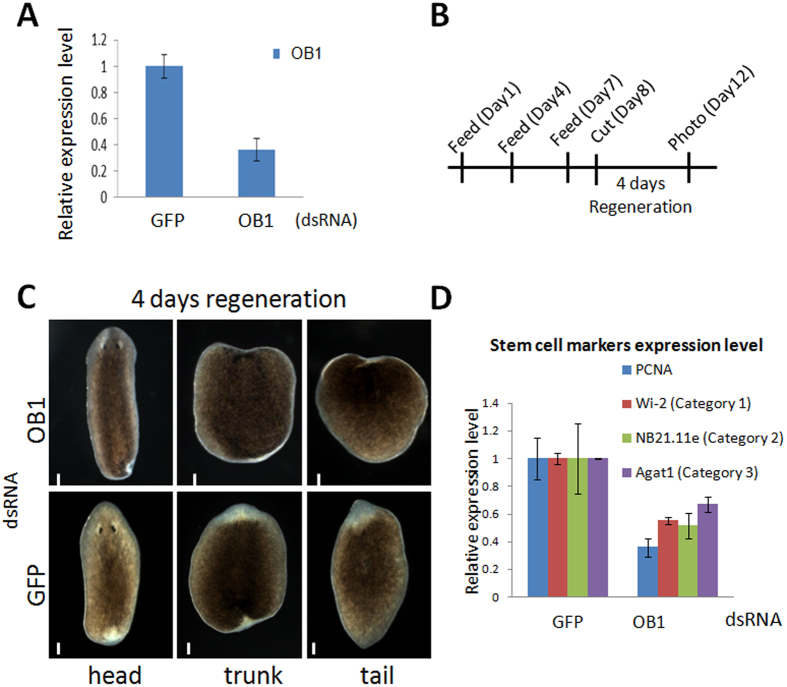
SmedOB1 is required for regeneration in *Schmidtea mediterranea.* (**A**) RNAi efficiency after one rounds of dsRNA feeding. (**B**) A flow diagram of the dsRNA-feeding and amputation assay. The day of cutting is indicated as Day 0. (**C**) The head, trunk and tail fragments were regenerated 4 days post-amputation. Phenotypic data were collected using a Zeiss SteREO Discovery V.20. Each group included 5 worms. One representative worm is shown. Scale bar: 1 mm. (**D**) q-PCR of the planarian proliferation markers and adult stem cell markers PCNA, Wi2, NB21.11e and Agat-1 in control and SmedOB1-RNAi planarians.

We next evaluated the effect of SmedOB1 on regeneration post-amputation. We first treated the worms with one round of dsRNA feeding. This treatment led to a 60% reduction in the SmedOB1 transcription level (Fig. 3A). The worms were then cut crosswise into three sections and further cultured (Fig. 3B). Approximately 4 days post-amputation, the control worms grew new white blastemas around the cut areas in each section, indicating successful tissue regeneration. However, in the SmedOB1-RNAi worms, there was a significant delay in the appearance and the extent of the blastema growth. This delayed regeneration phenotype was observed in all three sections (the head, trunk and tail) and was repeatable in different dsRNA feeding groups (Fig. 3C and Supplementary Fig. S3B). The above findings indicate that SmedOB1 is involved in the tissue regeneration process in planarians.

The stem cells in planarians can be divided into three categories based on the expression of different stem cell markers. Expression of Wi2 represents the early stage, high expression of NB21.11e represents the middle stage, and expression of Agat-1 represents the late stage^17^. All three stem cell markers were downregulated in planarians after SmedOB1 knockdown (Fig. 3D). Notably, the proliferation marker PCNA was also downregulated in these worms. These findings point to a reduced number of stem cells in SmedOB1 RNAi worms, possibly due to the decreased proliferation of these cells.

### SmedOB1 is required for telomeric DNA-protein complex association

**Figure 4:**
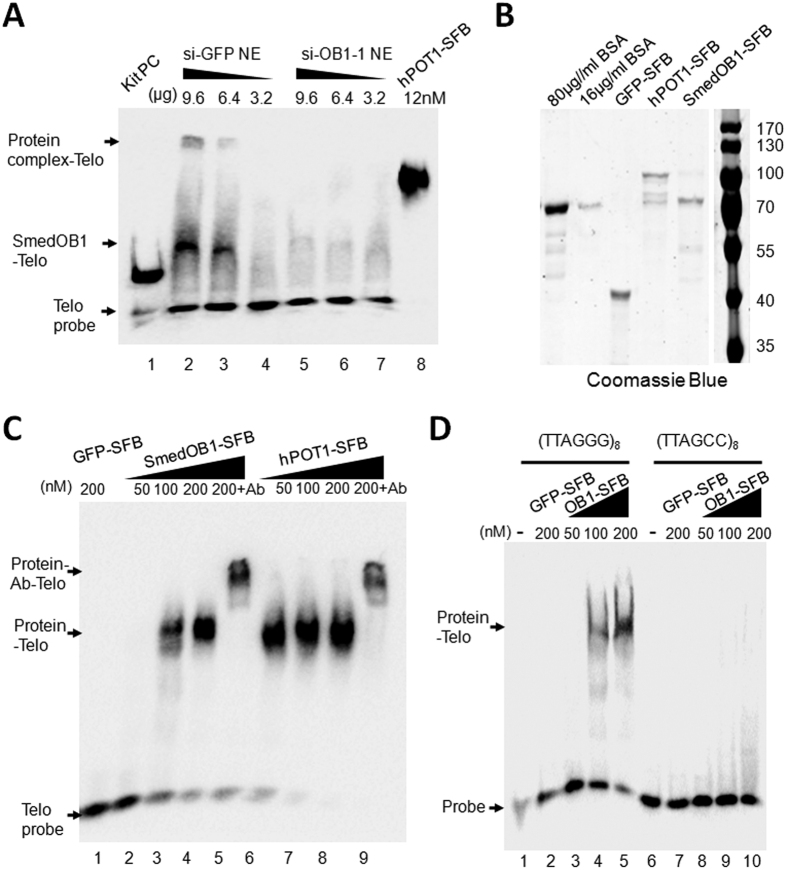
SmedOB1 is required for telomeric DNA-protein complex association. (**A**) Nuclear extracts (NE) were obtained from si-GFP and si-SmedOB1 planarian cells. EMSA assay using indicated amounts of NE and 1 nM (TTAGGG)_8_ probe (lanes 2–7). Lane 1, positive control provided with the kit; lane 8, 12 nM purified human POT1 (final concentration), two arrows indicated shifted bands (upper and middle) and the lower arrow indicated the free probe. (**B**) The C terminal SFB tagged GFP, human POT1 and SmedOB1 were expressed in 293T cells and purified with SBP tag. Coomassie Blue staining of purified GFP-SFB, human POT1-SFB and SmedOB1-SFB. BSA proteins were used as controls. (**C**) Electrophoresis mobility shift Assay (EMSA) for the full-length SmedOB1 with 1 nM (TTAGGG)_8_ ssDNA probe. Lane 1: 200 nM GFP-SFB; Lanes 2–4: SmedOB1-SFB (50 nM–200 nM); Lane 5: SmedPOT1-SFB (200 nM) plus FLAG antibody; Lanes 6–8: human POT1-SFB (50 nM–200 nM); Lane 9: human POT1-SFB (200 nM) plus FLAG antibody. Human POT1 was used as a positive control, and GFP was used as negative control. All the amounts refer to final concentration. The upper arrow indicated the supershift, the middle arrow indicated the Protein-ssDNA shift and the lower arrow indicated the free probe. (**D**) EMSA for the full-length SmedOB1 with 1 nM (TTAGGG)_8_ telomere probe (Lanes 1–5) or (TTAGCC)_8_ mutant probe (Lanes 6–10). Lanes 1 and 6, no protein; Lanes 2 and 7: 200 nM GFP-SFB; Lanes 3–5, 50 nM, 100 nM and 200 nM SmedOB1-SFB (OB1-SFB).

Next, we tested whether SmedOB1 could also bind single-stranded telomere DNA. We prepared the nuclear extract (NE) from planarians that were fed with one round of dsRNAs targeted against GFP or SmedOB1. By the same gradient, the si-GFP NE shifted the probe while the si-OB1 NE was unable to shift the telomere probe (Fig. 4A, lanes 2–4 compared to lanes 5–7). Interestingly, there are two shifted bands in si-GFP NE group. The upper shifted bands might be a multi protein-telomere complex while the lower shift indicates as SmedOB1-telomere complex (Fig. 4A, upper and middle arrows). These results suggested that SmedOB1 may be functionally similar to human POT1 and is required for protein-telomere complex formation. Next, SFB tagged full length SmedOB1 and human POT1 were purified from eukaryotic cells (Fig. 4B) and incubated with a biotin-labelled telomere probe (TTAGGG)_8_ (Fig. 4C). As shown in Fig. 4C, the human POT1 shifted the probe (Fig. 4C, lanes 6–8). With an increased gradient, the full length SmedOB1 was able to shift the oligo probe, although the signal is relatively weaker than human POT1 (Fig. 4C, lanes 2–4). SFB-tagged GFP purified from human 293T cells was used as a negative control. To check the specificity of this result, FLAG antibody which could recognize the FLAG in the SFB tag was used for supershift reactions. FLAG antibody retarded the protein-ssDNA complex to a higher molecular weight, for both human POT1 and SmedOB1, suggesting successful supershifts (Fig. 4C, lanes 5 and 9). Furthermore, a (TTAGCC)_8_ mutant probe was used for incubation with the SmedOB1 proteins, no binding could be detected. suggesting the specificity of its telomere binding (Fig. 4D). Collectively, these results confirmed that SmedOB1 is required for the telomeric protein-DNA complex formation and it associates with telomere (TTAGGG)_8_ ssDNA *in vitro*.

### SmedOB1 participates in the DNA damage response and cell survival

**Figure 5:**
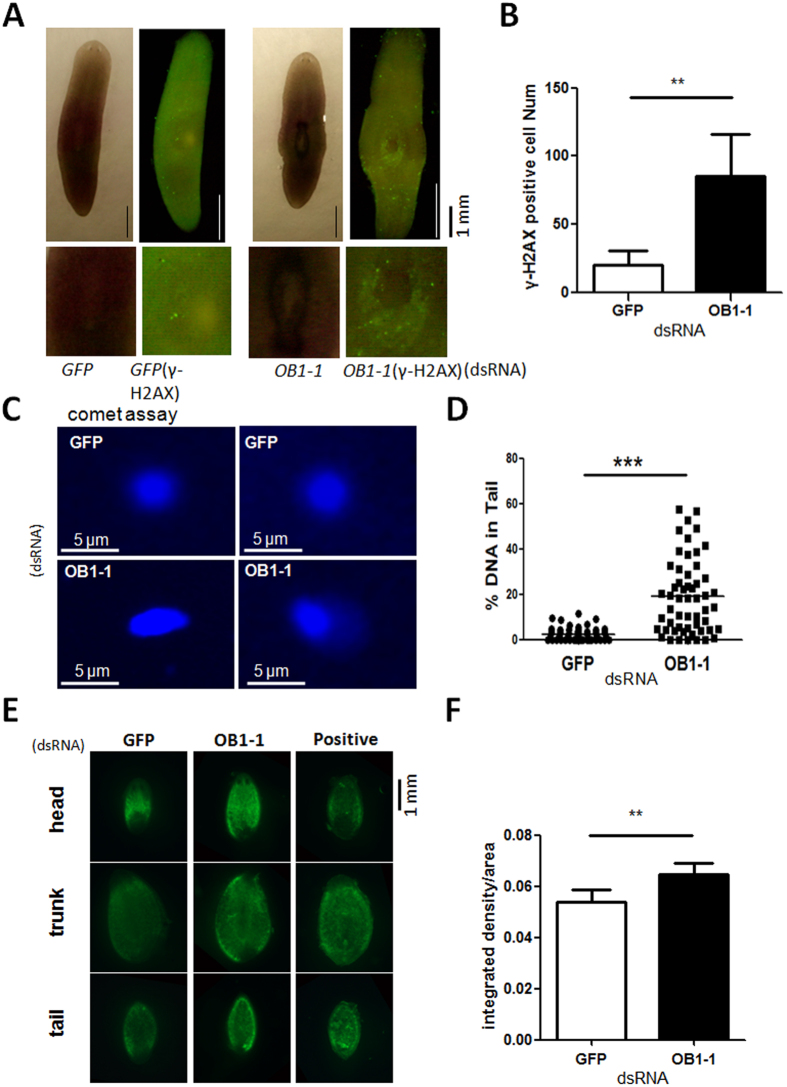
SmedOB1 RNAi significantly increases DNA damage and cell apoptosis. (**A**) Intact *Schmidtea mediterranea* planarians were fed GFP or SmedOB1 dsRNAs for two rounds and were subjected to whole-mount immunofluorescence using γ-H2AX. (**B**) The quantification of γ-H2AX was analysed in ImageJ software. (**C**) Alkaline comet assay was performed using single cells from planarians fed with GFP or SmedOB1 dsRNAs. (**D**) The quantification of alkaline comet assay was analysed in CASP software. (**E**) *Schmidtea mediterranea* planarians were fed GFP and SmedOB1 dsRNAs for two rounds and were then amputated. After regeneration for 8 days, the planarians were whole-mount stained with an *in situ* Cell Death Detection Kit, Fluorescein (Roche) and DAPI. Planarians treated with DNase I were used as a positive control. (**F**) The quantification of ΤUNEL was analysed in ImageJ software.

POT1 is important for controlling telomere length and protecting telomere ends. The loss of human POT1 results in DNA-damage responses at the telomere ends, telomere lengthening, cell cycle arrest, and cell death^18^,^19^,^20^,^21^. To better understand the role of SmedOB1 in these biological processes, we performed RNAi experiments in intact *Schmidtea mediterranea* (Fig. 2B,C). Then we performed the whole-mount immunofluorescence analysis using an antibody against the DNA damage marker γH2AX. The SmedOB1 RNAi planarians exhibited a significantly increase in γH2AX signals compared to the GFP RNAi control worms (Fig. 5A,B). In fact, these were the same areas that exhibited the tissue injuries (Fig. 2D). To further confirm this, we performed an alkaline comet assay using single cells from SmedOB1 RNAi and control planarians. Alkaline comet assay detects both single and double-strand DNA breaks. The SmedOB1 RNAi planarian cells showed significantly more comet tails than GFP RNAi cells (Fig. 5C,D). These results indicated that SmedOB1 likely plays a role in DNA damage response pathways.

The apoptosis signal is stimulated in amputated worms^22^. We next amputated the RNAi-treated worms and performed TUNEL assays. After regeneration, we detected a significant increase in the apoptotic signals in the SmedOB1-RNAi planarians compared to the control group (Fig. 5E,F). These results indicate that SmedOB1 regulates planarian homeostasis by inhibiting DNA damage and cell apoptosis.

### Functional identification of DjOB1 in the planarian *Dugesia japonica*

**Figure 6:**
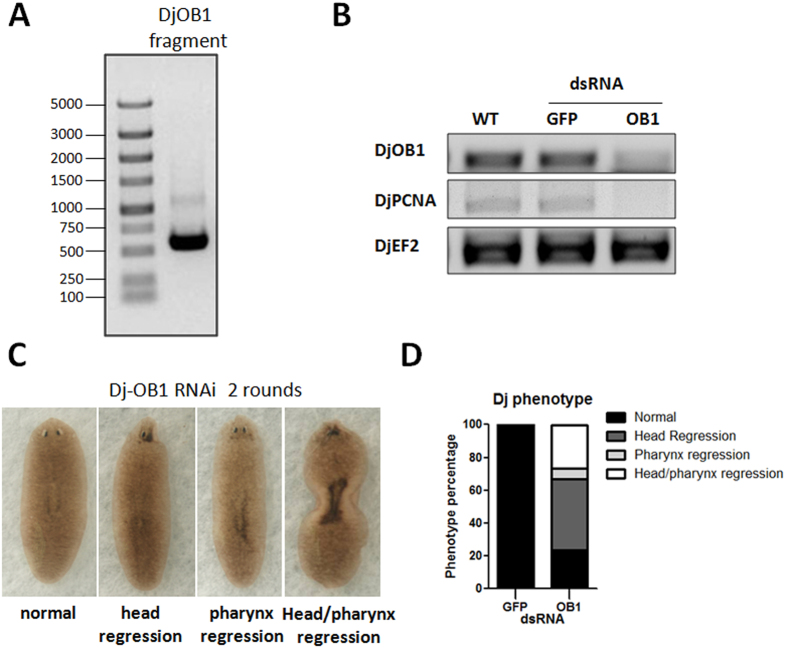
Functional identification of DjOB1 in planarian *Dugesia japonica*. (**A**) PCR cloning of a partial DjOB1 sequence base on Unigene 34129. (**B**) RT-PCR analysis after two rounds of dsRNA feeding. DjPCNA and DjOB1 were analysed, and DjEF2 was used as an internal control. (**C**) Phenotypes of *Dugesia japonica* after two rounds of dsRNA feeding. (**D**) Statistical quantification of the proportion of abnormal flatworms in *Dugesia japonica* after RNAi DjOB1. The planarians with injuries on the head, pharynx or both were analysed.

The planarian *Dugesia japonica* also contains a TTAGGG telomere repeat sequence (Supplementary Fig. S1). Therefore, we investigated whether OB1 is conserved in the planarian species *Dugesia japonica (Dj).* A database search and analysis identified one Unigene 34129^23^,^24^, and we used PCR to clone a 508-bp partial DjOB1 sequence. We then generated dsRNAs based on this sequence (Fig. 6A) and conducted RNAi experiments in *Dugesia japonica*. After feeding, DjOB1 levels were dramatically reduced (Fig. 6B). Similar to the SmedOB1 RNAi worms, we observed decreased expression of the proliferation marker PCNA (Fig. 6B), and most of the DjOB1-RNAi *Dugesia japonica* showed tissue injuries to the head eyespots, pharynx or both, whereas the GFP-control-treated worms were completely normal (Fig. 6C,D). These results suggest that the planarian OB1 gene is functionally conserved among planarian species.

## Discussion

Pot1 is an essential gene, but deletion or mutation of Pot1 is associated with various phenotypes in different species. For example, deletion of Pot1 in S.pombe leads to rapid telomere loss, while the inhibition of POT1 in humans increases telomere length^8^. Thus, when we identified a POT1 homologue in planaria called SmedOB1, it was of interest to determine whether SmedOB1 regulates telomeres in planaria and to examine the phenotypes of planaria treated with RNAi. Our results showed that the lack of SmedOB1 in the flatworm caused tissue injuries and regeneration defects at the individual level. Thus, SmedOB1 is required for planarian homeostasis and regeneration. Mechanistically, we hypothesized SmedOB1 may regulate ASC function by promoting ASC proliferation, and inhibiting DNA damage and cell apoptosis in a telomere-dependent manner.

The expression pattern of SmedOB1 indicated that all of the cells in a planarian express the OB1 gene, but the RNAi phenotype was first observed in the areas around the pharynx and around the eyespots, where fewer ASCs are present^17^. We hypothesized that this difference occurred because when tissue is injured, the planarian ASC pool dispatches stem cell progenies to migrate to the injured locations, where they complete division as well as differentiation. Because the areas around the pharynx and the eyespots have few ASCs, injuries around these areas cannot heal quickly. SmedOB1 expression is not restricted to ASCs but is ubiquitous and observed in both ASCs and somatic cells; thus, it is important for whole-body homeostasis.

The planarian flatworm is an ideal model for studying regeneration and stem cells, and many hypotheses that were previously based on the findings of *in vitro* experiments can be confirmed using *in vivo* experiments in planaria. Further, because planaria have the same telomere repeat sequence as humans, we believe that planaria will also be suitable for telomere studies. We studied SmedOB1 in planaria because its sequence is conserved among all of the shelterin complexes, while no other telomeric proteins could be predicted in planarians through BLAST analysis of conserved domains. Here we found that SmedOB1 associates with the telomere TTAGGG sequence *in vitro* and is required for planarian homeostasis and regeneration. The phylogenetic and functional conservations of the SmedOB1 provide one of the mechanisms by which planarians maintain telomeres and genome stability to ensure their immortality. Interestingly, we also found that besides SmedOB1 itself, there may be a potential multi protein-telomere complex in planarian, suggesting other un-identified telomere associating proteins existing in this species (Fig. 4A). Further studies elucidating these proteins or complexes would make great efforts to this field.

SmedOB1 is the first telomere-associated protein identified in planaria so far. The telomere structure is complicated and the maintenance of telomere structure, length and function require multiple proteins as well as TERRA^5^. By using high-throughput biochemical methods such as proteomics of isolated chromatin segments (PiCH), DNA-protein affinity assays and quantitative proteomics, unknown planarian telomeric proteins will be identified in the near future. A complete telomeric protein network will provide additional insights regarding telomere regulation in planarian adult stem cell biology. Further studies will shed light on the regeneration medicine of humans.

## Methods

### Planarian cultures

The asexual strain of *Schmidtea mediterranea* was maintained in 1X Montjuic water at 20 °C as previously described^25^. A clonal line of the mixoploid asexual strain of *Dugesia japonica* was derived from a single worm collected from Beibei Mountain in Chongqing and was maintained in filtered tap water at 22 °C. The worms were fed with beef liver once per week and starved for at least one week prior to all of the experiments. For irradiation, the planarians were exposed to X-rays at 5 Gy or 10 Gy and then maintained in culture medium for 4 days before RNA extraction.

### Identification and cloning of SmedOB1 and DjOB1

A BLAST-based reciprocal best-hit method, in combination with protein sequence alignment, was used to identify orthologous POT1 genes in planarians as previously reported^25^. Briefly, the human POT1 isoform 1 was used to perform a tBLASTn search in the *Schmidtea mediterranea* genome database SmedGD 2.0 (http://smedgd.stowers.org) with an e-value of 1e-08. The genomic sequence (V31.002347: 3945..3613) was then translated and compared with human sequences using NCBI BLAST tools.

To clone SmedOB1, RACE primers were designed accordingly, and 5′ RACE and 3′ RACE were performed using the FirstChoice® RLM-RACE Kit (Thermo Scientific). The PCR products were TA-cloned using the pEASY-T1 vector (Transgene) for sequence verification. The following primers were used: 5′RACE inner primer FP: ATCCTAgggAgATTTTCAgg; 5′ RACE gs primer RP: AAATACAGCCACGTTGAATCCT; 3′RACE outer primer: GATTTTACTGCTTTAACAGATG; 3′RACE inner primer: AGGATTCAACGTGGCTGTATTT. A partial *Dugesia japonica* POT1 homologue (DjOB1) was cloned by PCR using the following primers. DjOB1 FP: 5′-ATTACAAAGACAAGCCTCAG-3′; DjOB1 RP: 5′-GAAAATAAACATAATCTCCTGG-3′.

### DsRNA-mediated knockdown in planarians

DsRNAs for gene knockdown were *in vitro* transcribed using the mMESSAGE mMACHINE T7 kit (Ambion). The PCR templates were generated with T7 promoter sequences at both ends and were then fed to the worms as previously described^26^. Ten micrograms of dsRNAs was used for every ten worms. GFP dsRNAs were used as negative controls. Three different dsRNAs that targeted different regions of SmedOB1 were employed. The following primers were used: SmedOB1–1 FP-T7: taatacgactcactatagggTACGATTTTACTGCTT; SmedOB1-1 RP-T7: taatacgactcactatagggAAATACAGCCACGTTG; SmedOB1-2 FP-T7: taatacgactcactatagggTCAAAAAACTATTTGATAGATACAT; SmedOB1-2 RP-T7: taatacgactcactatagggTTCAAGTCTTGCGAGTAA; SmedOB1-3 FP-T7: taatacgactcactatagggTGATAAATTACTCGCAAGAC; SmedOB1-3 RP-T7: taatacgactcactatagggTTCCCTTCAATCCATCC. For one round of feeding, the worms were treated three times in one week (days 1, 4 and 7, Fig. 3B). For two rounds of feeding, the worms were treated at different time points during a three-week period (day 1, 4, 7, 14, 17 and 20, Fig. 2B).

### Whole-mount *in-situ* hybridization (WISH)

WISH experiments were performed as previously described^25^. *In situ* hybridization of the telomeres was performed using a metaphase spread and a PNA-TelC-FITC probe (Panagene) as previously reported^14^. Visualization was performed on the Zeiss SteREO Discovery V.20. The WISH probes were produced using the DIG RNA Labelling kit (T7/SP6) (Roche). The probe templates were produced by PCR with a T7 promoter sequence added to the 5′ end. The antisense probes for SmedOB1 and Smedwi-1 were generated with the following primers: SmedOB1 FP: AAGGAATGTTTTGAACG; SmedOB1 RP: TAATACGACTCACTATAGGGTACTGCTGACTTTCCTT. Smedwi-1 FP: TACAGCCTGATACAGTTAC; Smedwi-1 RP: taatacgactcactatagggTTGTAGTAGAATACCCTCC.

### Quantitative PCR

RNA was extracted with TRIzol (Invitrogen) and reversed transcripted with the iScript cDNA Synthesis kit (Bio-Rad). q-PCR was performed using the GoTaq qPCR kit (Promega). The primers are listed below. Cystatin was used as the internal reference. Smedwi-2 qFP: CTGCAAAAGATTACCTATGCTCTAACT; Smedwi-2 qRP: ACGGGATTAGAGCCCTTATGCAC; SmedNB.21.11e qFP: GTCTCCCGCCAAATCAAGTA; SmedNB.21.11e qRP: TTTCATGCAATCTGCTTTCG; SmedAgat1 qFP: GTTGGTTGAAAAGCGAGAGGTT; SmedAgat1 qRP: CGAACATCGGAAGTCCAACAATG; SmedCystatin qFP: AACTCCATGGCTAGAACCGAA; SmedCystatin qRP: CCGTCGGGTAATCCAAGTACA; 

SmedOB1 qFP: TGTTGAACCCCCTCTTAACG; SmedOB1 qRP: GATGCAATCAGCTACGCAAG. DjEF2 FP: 5′-GCAATCGAAGACGTTCCATGTG-3′; 

DjEF2 RP: 5′-CCAGGAAAAGTTGTTATAGTCCCAGTTT-3′; DjPCNA FP: 5′-ATGCCTTGGAAGCAATGATTCTTTAACAAT-3′; DjPCNA RP: 5′-TAAATGGTCTCCCTCTATATCCATCAATTT-3′; DjOB1 FP: 5′-GAAATTCAATGCCATCTTCAGAG-3′; DjOB1 RP: 5′-CACAAGCAGGAACAGTACCGTC-3′.

### Protein purification and electrophoretic mobility shift assay (EMSA)

Nuclear extracts (NE) were obtained from planarians after one round of dsRNA feeding and starvation for one week, using NE-PER Nuclear and Cytoplasmic Extraction Reagents (Thermo Scientific). 293T expressed SFB-tagged full length human POT1 and SmedOB1 were purified using High Capacity Streptavidin Agarose (Thermo Scientific) and 2 mM Biotin (Sigma-Aldrich) elution, verified by SDS-PAGE, and then incubated (at different concentrations) with a biotin-labelled telomeric single-stranded DNA probe (TTAGGG)_8_ or mutant probe (TTAGCC)_8_ (Synthesized in Thermo Scientific) at room temperature for 30 minutes. The reactions were then resolved by electrophoresis and blotted for biotin detection using the Chemiluminescent Nucleic Acid Detection Module (Thermo Scientific). For supershift, FLAG antibody (Abmart) was used at a final concentration of 50 μg/ml.

### Whole-mount immunostaining and comet assay

IF was performed as previously reported^25^. In brief, fixed and bleached worms were rehydrated in graded PBSTx (PBS + 0.3% Triton X-100)/methanol solutions, blocked in PBSTx containing 0.25% BSA (Sigma-Aldrich), and incubated with primary antibodies overnight at 4 °C (γ-H2AX, Millipore). After extensive washing with PBSTx, samples were incubated with Alexa Fluor 488-conjugated secondary antibody and mounted using fluorescent mounting medium (Dako). Alkaline comet assay was performed according to the manufacturer’s protocol (Trevigen). The DNA was stained by Gel-Red (Beyotime) and photographed in Zeiss SteREO Discovery V.20. The quantification of data was analysed in CASP software.

### TUNEL assay

TUNEL assays using the planarians were performed as described previously^25^. Briefly, the worms were sacrificed in 10% n-acetyl cysteine (Sigma), fixed in 4% formaldehyde, and permeabilised in 1% SDS (for 20 min) before being bleached overnight in 6% H_2_O_2_ (diluted in 1x PBST). Following the washes in 1xPBST and 1xPBS, the worms were incubated with the terminal transferase enzyme (Roche) for 1 h at 37 °C, and worms treated with DNase I (NEB) for 10 min at RT were used as positive controls. The planarians were incubated with DAPI for 10 min before mounting. The signals were photographed in Zeiss SteREO Discovery V.20. The quantification of data was analysed in ImageJ software.

### Data analysis

All the statistic data are presented as means ± SDs. The statistical significance of differences between two means was calculated using Student’s t-test. The probability level accepted for significance was P < 0.05 (*) and P < 0.01 (**).

## Additional Information

**How to cite this article**: Yin, S. *et al.* SmedOB1 is Required for Planarian Homeostasis and Regeneration. *Sci. Rep.* **6**, 34013; doi: 10.1038/srep34013 (2016).

## Supplementary Material

Supplementary Information
