# Supplementary material for: SmedOB1 is Required for Planarian Homeostasis and Regeneration
Source: Sci Rep. 2016 Sep 22;6:34013. doi: 10.1038/srep34013 (PMC5032016; doi:10.1038/srep34013)
Supplement: Supplementary Information [file srep34013-s1.pdf]

Supplementary Information for

SmedOB1 is Required for Planarian Homeostasis and Regeneration

Shanshan Yin [1, #], Yan Huang [1, #, *], Yingnan Zhangfang [1], Xiaoqin Zhong [1], Pengqing Li [1], Junjiu Huang [1], Dan Liu [2] and Zhou Songyang [1, 2]

[1] Key Laboratory of Gene Engineering of the Ministry of Education, and State Key Laboratory of Biocontrol, School of Life Sciences, Sun Yat-sen University, Guangzhou, China; [2] Verna and Marrs McLean Department of Biochemistry and Molecular Biology, Baylor College of Medicine, Houston, USA

*Correspondence should be addressed to Y.H. (huangy336@mail.sysu.edu.cn).

# These authors contributed equally to this work.

Fig. S1

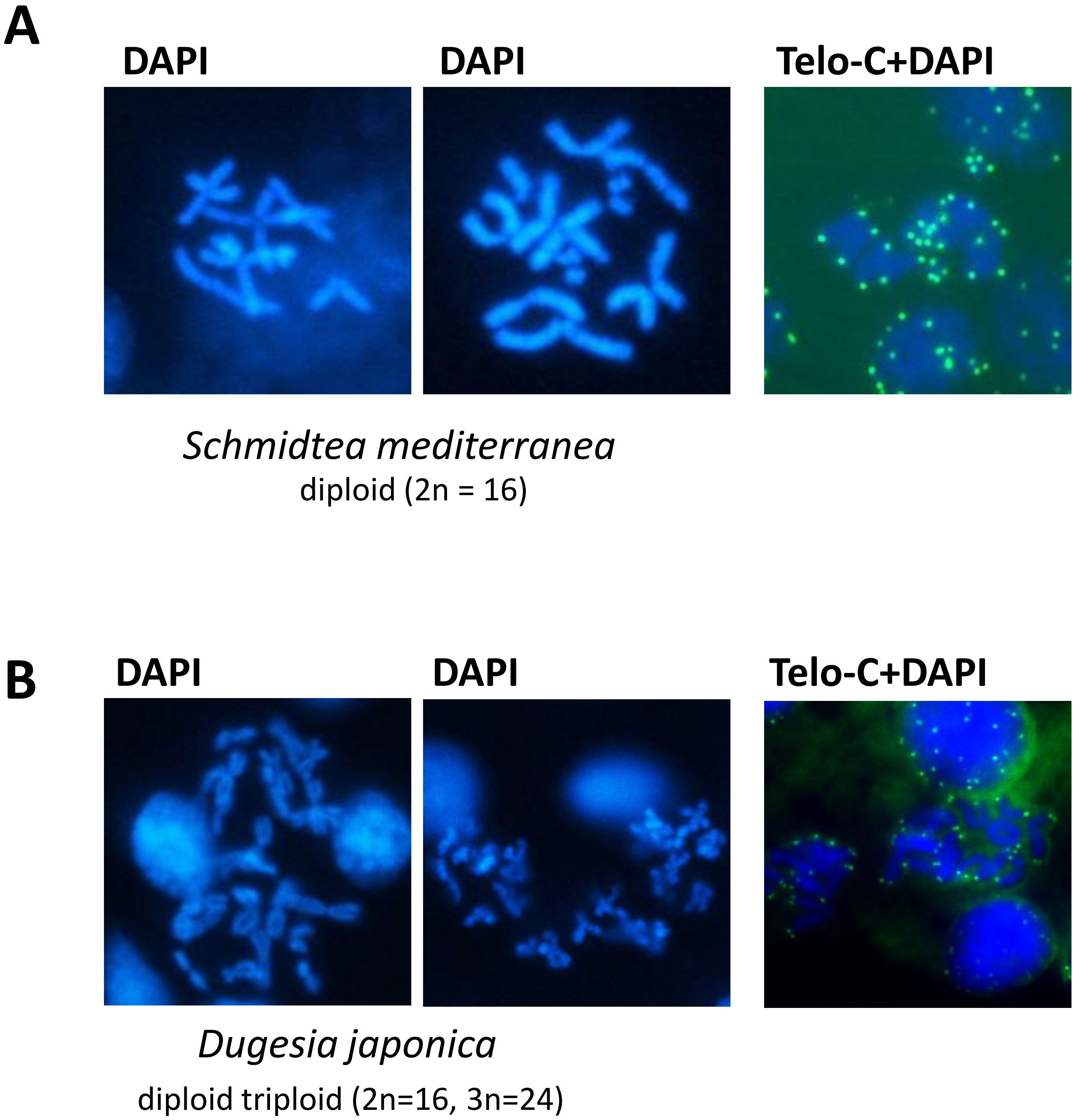

Fig. S1 Metaphase karyotype analysis of planarians *Schmidtea mediterranea and Dugesia japonica.* (A) *Schmidtea mediterranea* Metaphase karyotype and FISH for telomere probe $(TTAGGG)_8$.

Fig. S2

A SmedOB1 nucleotide sequence

>ATGCAATACGATTTTACTGCTTTAACAGATGTTAAAGTTGGTGAATTTTCCAATGTCATGGG
TATAATAAAATATTGTTCTGATCCAAAAAAGACAAAGGGTAAAGATTACCAAATGACTGTAAG
CATAACTGACGCATCTGTTGGATATGAAGGGAAAATCGTGTGCAATCTTTTTAATGCAAAAC
CTGAAAATCTCCCTAGGATTACTGGAAGAGGAGATGTTATAATAATGCACAAATTAAAAATAA
GCAAATTTAGAAATAACATTCAAGGAATAGGAATTCAAAAAGAAGGATTCAACGTGGCTGTAT
TTTCAAAAAACTATTTGATAGATACATCAAAGGAATGTTTTGAACGCATAATCGCCAACATGA
GTTCAATAAAAAATCAGTTCTCTATTACAATGGATGATTTAAAAAAGGTTCAAAAACTTCAAG
AATGGGGAAATTCAATGCCTTCTTCACAAATTGATGAAGGCGAATTAAATCTGGATGCAATAA
ACTTTGTTTCGTTGTCAGAAATCAGCCCTAGAGTTACATGCTCGTTGACAGCACAAGTTATC
AGTGTTCTTTCTATAAGCTATCTCGATTTTCGAACGTTAATTTTGATCTGGGATGGTACTATTC
CCGGATGTGATCTCAACAGAAAGTTCATCACTATCCTTTCGAATTGCATTAAAGAAAAACAC
GATACAGAGCTGATGGCCCAATTCGGGGGCAAAATATCTAATCCATATCTGGGTTATATTTTC
TTGAAAGAAGAATCATTAATCGGTCCCGGTGACTATGTGTACTTTCATAGGGTTTTTGTTGA
ACCCCCTCTTAACGGTCCAGGAGTTTGTCTTGTCGCAGATCGATGCAAGGTTGGAATATTG
ACGAAGGAAAGTCAGCAGTATGATAAATTACTCGCAAGACTTGAAAGTTTCTCTGAAAGCTT
ACCTTGCGTAGCTGATTGCATCAGTATTGATTTAAAACAACTGACTAAATGGTCACTAAAAGA
TTACGGGAAAATGCTGAAACAATTTGATTATGAAATTGAATTCAATGAGAATGAAAAATATCAA
TTTAATATAACTGCACGAGTAGTGAAGGTTCATCCAAGCACAGAAAAAGAATTCAATGATTC
GATGGTCATATTTTGCTCGTGCTGCCAAGAATTGAGAGATATGAAAGATGTTTCACAATTGG
CATATGCTTGCCCAAAGTGTGCTGTGCCTATTCCTATGATTACAATGGTATTTGAGGATTTCA
CAGGATGGCTGACTGGTGTTGTTGTCGGTCAGTCTGTGTTGGATATGTTGAATCTGGTAAT
GATCGATGCCAAGCAACCGAACTGGTGGGTGCACTTGTTTGGTCAGAATAAAATCGAGTCA
ATAAATCAGTGGCTTTCCCATGTCAAGCGATTAGCTGATGGATGGATTGAAGGGAAATTGGT
TTTCAAACGGACCCTTCACGGAAAAGTTTTACCATTTATCGAAGATATTGTTTTATTGGATTAT
TTATAA

B SmedOB1 protein sequence

>MQYDFTALTDVKVGEFSNVMGIIKYCSDPKKTKGKDYQMTVSITDASVGYEGKIVCNLFNAKP
ENLPRITGRGDVIIMHKLKISKFRNNIQGIGIQKEGFNVAVFSKNYLIDTSKECFERIIANTSSIKNQ
FSITMDDLKKVQKLQEWGNSMPSSQIDEGELNLDAINFVSLSEISPRVTCSLTAQVISVLSISYLD
FRTLILIWDGTIPGCDLNRKFITILSNCIKEKHDTELMAQFGGKISNPYLGYIFLKEESLIGPGDYV
YFHRVFVEPPLNGPGVCLVADRCKVGILTKESQQYDKLLARLESFSESLPCVADCISIDLKQLTK
WSLKDYGKMLKQFDYEIEFNENEKYQFNITARVVKVHPSTEKEFNDSMVIFCSCCQELRDMKD
VSQLAYACPKCAVPIPMITMVFEDFTGWLTGVVVGQSVLDMLNLVMIDAKQPNWWVHLFGQN
KIESINQWLSHVKRLADGWIEGKLVFKRTLHGKVLPFIEDIVLLDYL

Fig. S2 Assembly of 5’ RACE and 3’ RACE PCR results showed full length SmedOB1 sequence. (A) SmedOB1 nucleotide sequence. (B) SmedOB1 predicted protein sequence.

Fig. S3

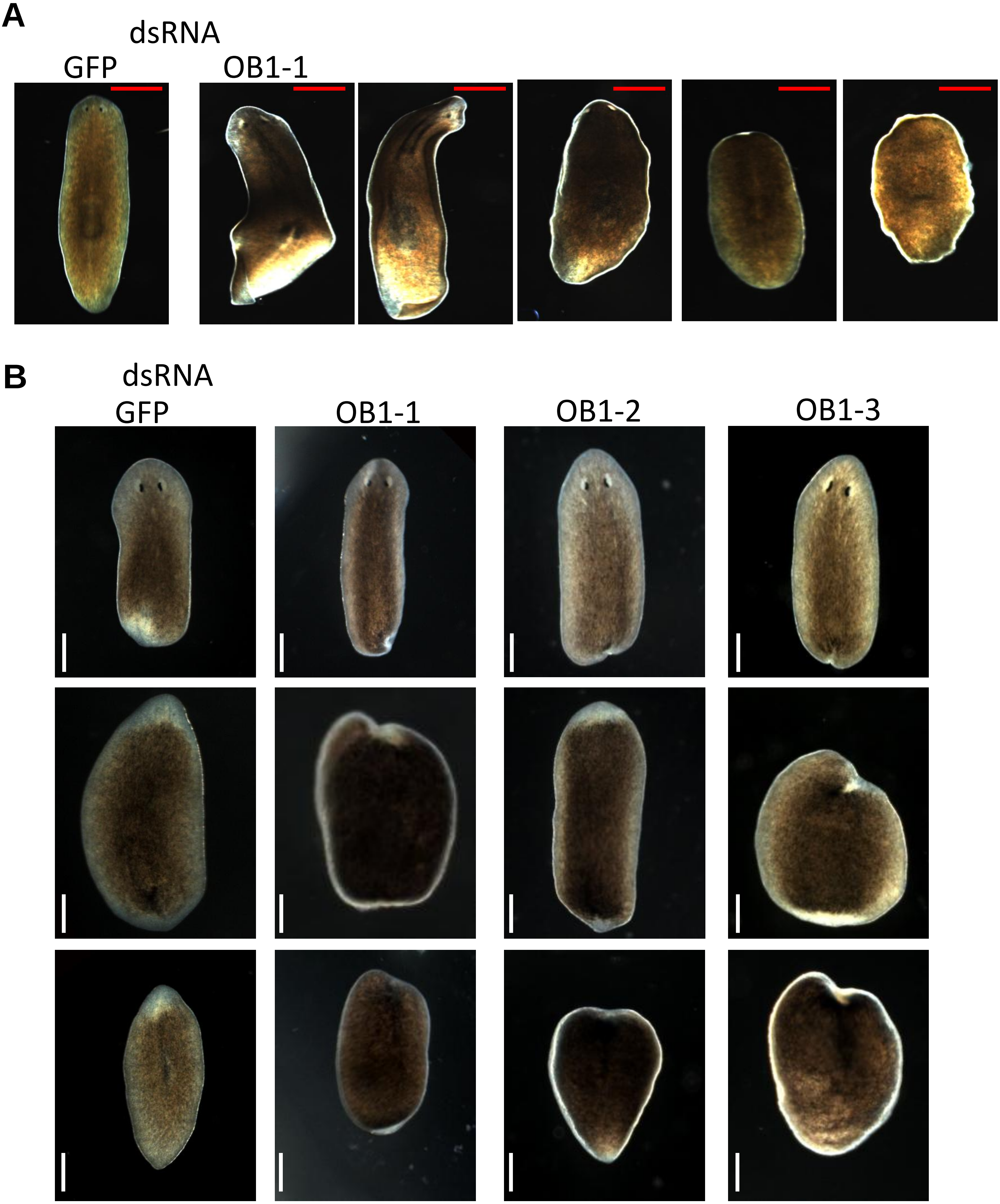

Fig. S3 Phenotype of *Schmidtea mediterranea* after SmedOB1 dsRNA feeding for 2 rounds (A) and regenerating for 4 days postamputation. (A) Left: GFP dsRNA control; right: SmedOB1 dsRNA. Scale bar, 1mm. (B)) Left: GFP dsRNA control; right: SmedOB1-1, SmedOB1-2, SmedOB1-3 dsRNA. Scale bar, 1mm.

Fig. S4

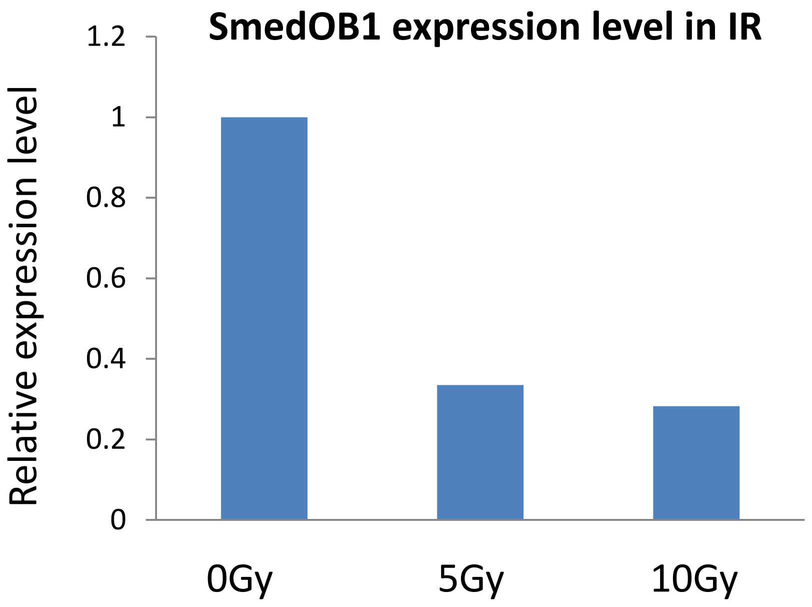

Fig. S4 SmedOB1 expression in *Schmidtea mediterranea* before and after irradiation. The planarians were exposed to 5Gy or 10Gy X-ray and then maintained in culture medium for 4 days before RNA extraction.
